# Low Holding Densities Increase Stress Response and Aggression in Zebrafish

**DOI:** 10.3390/biology11050725

**Published:** 2022-05-09

**Authors:** Marica Andersson, Jonathan A. C. Roques, Geoffrey Mukisa Aliti, Karin Ademar, Henrik Sundh, Kristina Sundell, Mia Ericson, Petronella Kettunen

**Affiliations:** 1 Department of Psychiatry and Neurochemistry, Institute of Neuroscience and Physiology, Sahlgrenska Academy at the University of Gothenburg, 413 45 Gothenburg, Sweden; marica.andersson@gu.se (M.A.); geoffrey.aliti@gu.se (G.M.A.); karin.ademar@gu.se (K.A.); mia.ericson@gu.se (M.E.); 2 Department of Biological and Environmental Sciences, University of Gothenburg, 405 30 Gothenburg, Sweden; jonathan.roques@bioenv.gu.se (J.A.C.R.); henrik.sundh@bioenv.gu.se (H.S.); kristina.sundell@bioenv.gu.se (K.S.); 3 Department of Marine Sciences, University of Gothenburg, 405 30 Gothenburg, Sweden; 4 Swedish Mariculture Research Center (SWEMARC), University of Gothenburg, 405 30 Gothenburg, Sweden

**Keywords:** zebrafish, welfare, holding density, behaviour, husbandry, water cortisol

## Abstract

**Simple Summary:**

Zebrafish are used as experimental animals in labs all around the world. To ensure that the health of zebrafish is maintained at the highest level, it is important to know the optimal housing conditions of the animals, including the housing density. Guidelines for housing densities of zebrafish can then be spread and followed globally, making it possible to compare research data from different facilities. To investigate the optimal housing densities of zebrafish and to better understand how holding density affects zebrafish behaviour and physiology, we evaluated the welfare of zebrafish housed at different densities for nine weeks. We observed that fish housed at the lowest holding density of 1 fish/L stood out from the rest of the experimental fish, showing higher levels of aggression, secreting more of the stress hormone cortisol in the water, and spending more time in the top zone of the tank, possibly reflecting the fact that fish in this density were hiding more behind the floating plants. Our data indicate that zebrafish should not be kept at 1 fish/L, or lower, to ensure good welfare of the animals.

**Abstract:**

With laboratory zebrafish (*Danio rerio*) being an established and popular research model, there is a need for universal, research-based husbandry guidelines for this species, since guidelines can help promote good welfare through providing appropriate care. Despite the widespread use of zebrafish in research, it remains unclear how holding densities affect their welfare. Previous studies have mainly evaluated the effects of holding densities on a single parameter, such as growth, reproductive output, or social interactions, rather than looking at multiple welfare parameters simultaneously. Here we investigated how chronic (nine weeks) exposure to five different holding densities (1, 4, 8, 12, and 16 fish/L) affected multiple welfare indicators. We found that fish in the 1 fish/L density treatment had higher free water cortisol concentrations per fish, increased vertical distribution, and displayed aggressive behaviour more frequently than fish held at higher densities. On the other hand, density treatments had no effect on anxiety behaviour, whole-brain neurotransmitter levels, egg volume, or the proportion of fertilised eggs. Our results demonstrate that zebrafish can be held at densities between 4 and 16 fish/L without compromising their welfare. However, housing zebrafish in the density of 1 fish/L increased their stress level and aggressive behaviour.

## 1. Introduction

The zebrafish (*Danio rerio*) is becoming an increasingly popular research model around the world due to its favourable traits, such as low maintenance costs, short generation time [1], biological similarity to humans in certain aspects [2], and responsiveness to genetic manipulation [3]. It is utilised in numerous research fields, including developmental and molecular biology, evolution, neuroscience, biomedical research, and genetics [4,5]. As the use of laboratory zebrafish increases, their welfare has been brought into focus. Ensuring good welfare is important for ethical reasons, but it is equally important for the experimental outcome by improving the accuracy and reproducibility of the experiments [2].

Zebrafish are naturally distributed across freshwater habitats in the monsoon areas of Pakistan, India, Nepal, and Bangladesh [6,7]. The species is known to occupy a significant variety of habitats in terms of water flow, temperature, bottom substrates, and vegetation [6,7,8,9]. The large inter-populational variations in habitat and ecological context across the distribution area have led to a divergent selection of traits [7,10], including social behaviour [11], group cohesion [6], shoal size [6], boldness [10], aggression [9], and even morphological differences [7]. The large variation in life cycle and wild behaviour brings about the question of which physical parameters that are most critical for their welfare, such as water quality, temperature, or shoaling density.

Fish commonly have different density-dependent physiological, behavioural, and psychological processes throughout their life cycle, where stocking density can affect the outcome of, for example, reproduction, growth, and survival [12]. Different density considerations need to be made depending on the species and their biological and behavioural characteristics, such as predatory, cannibalistic, or schooling fish. Studies on various farmed fish species have found adverse effects when housing fish in suboptimal stocking densities, such as reduced growth and increased mortality in black sea turbot (*Psetta maxima*) [13], reduced immune response in ayu (*Plecoglossus altivelis*) [14], and reduced intestinal barrier function and elevated cortisol levels in salmonids [15,16]. These findings illustrate the importance of studying the holding densities of fish, and that the density response in zebrafish must be evaluated to create a healthy and low-stress laboratory environment for them. 

There are certain local recommendations for zebrafish holding densities, for example in Sweden, the Board of Agriculture’s Regulations and General Advice of Laboratory Animals states that “Fish that are less than 5 centimetres long, should have at least 1 litre of water per fish” (own translation) [17]. However, guidelines such as this do not consider the inter-species differences in social structure and behaviour that exist for small fish, and it is unclear what facts these recommendations are based on. Moreover, the Guide for the Care and Use of Laboratory Animals informs us that the most current holding density in the USA is 5 fish/L, but does not give any recommendations on holding densities [18]. Lidster and colleagues [19] performed a survey of 98 animal facilities worldwide and found the most commonly reported stocking density of zebrafish to be 1–5 fish/L while highlighting the lack of scientific literature on the subject. 

Thus, there is a lack of evidence-based guidelines for the holding density of zebrafish [3,5]. In our recent review, we found that studies on zebrafish holding densities had little consistency in terms of tested density ranges and exposure times, and identified a need for future studies to evaluate the long-term effects of higher holding densities above 1 fish/L [20]. Additionally, the review underlined the need to evaluate multiple welfare indicators in the same experimental set-up, since most studies so far have investigated only one at a time, for example, cortisol stress response or reproduction [20].

**Figure 1:**
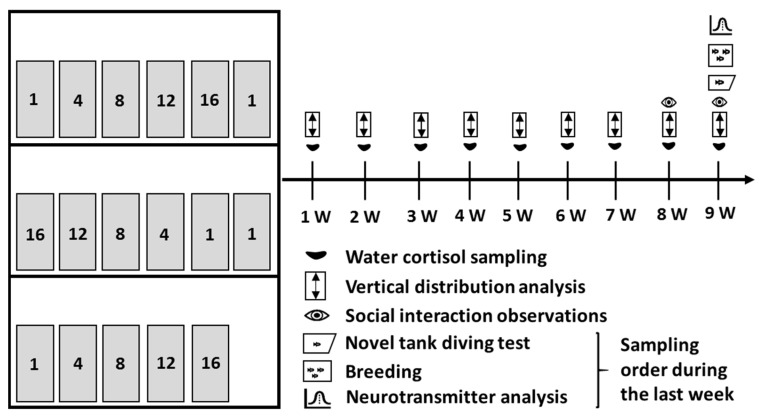
The experimental design. The left part of the figure illustrates the experimental set-up, and the right part of the figure illustrates the time frame of the experiment. For the set-up, each shelf held at least one tank from each density treatment. The densities were placed in increasing order from left to right on the top and bottom shelf, and from right to left on the middle shelf.

The aim of the present study was to examine how holding zebrafish at densities of 1, 4, 8, 12, and 16 fish/L, respectively, during a nine-week exposure period affects a number of welfare indicators. We chose to use a mix of both established and novel welfare indicators to assess zebrafish in different housing densities, as presented in Figure 1.

Throughout the entire exposure period, we performed two different non-invasive welfare assessments. Primarily, weekly water samples were taken from each exposure tank for cortisol analysis to assess stress levels. Secondly, the fish tanks were recorded for analysis of vertical distribution, which is a known change in response to stressors for other fish species [21,22,23]. Therefore, we wanted to evaluate if measurements of vertical distribution would be a useful non-invasive welfare indicator of stress and welfare in a density experiment with zebrafish. During the last exposure week, we also performed behavioural observations to investigate the effects on the social interactions of zebrafish in terms of chasing and hiding behaviour. Chasing is considered an aggressive behaviour associated with dominant zebrafish in hierarchical social structures which causes subordinates to retreat and hide [24,25]. We also performed the novel tank diving test to evaluate the anxiousness of the fish, detected by increased vertical exploration in a new fish tank over time [26,27,28,29,30]. The reproductive output of zebrafish is an important consideration for most zebrafish holding facilities and we, therefore, evaluated that as well. Lastly, we measured whole-brain monoamine levels of dopamine, serotonin, and norepinephrine, since they are connected to the zebrafish’s behavioural output and cognitive function, and altered levels are indicators of chronic stress and anxiety [31,32,33,34,35]. We considered the combination of these behavioural and physiological indicators to give a comprehensive assessment of how different holding densities affect zebrafish welfare.

## 2. Materials and Methods

### 2.1. Experimental Set-Up

In this study, 374 wild type zebrafish were obtained from the zebrafish core facility at Karolinska Institutet, Stockholm, Sweden, at three months of age. At Karolinska Institutet, the fish had been held at a density of 5 fish/L, which was maintained for one month of acclimatisation after their arrival at the Department of Biology and Environmental Sciences, University of Gothenburg, Gothenburg (Sweden). Thereafter, the fish were divided into 17, 3.5 L standard plastic tanks with dimensions of 25 × 10 × 12 cm (L × W × H) (Techniplast, Buguggiate, Italy), filled with 3 L of water, at five different holding densities of 1, 4, 8, 12, and 16 fish/L, respectively. We used 3 L tanks to ensure that our results would be of relevance since 3 L tanks are commonly used in zebrafish laboratories, holding fish in recirculating systems. There were three tanks for each density and five for the lowest density. We had two additional tanks for the 1 fish/L treatment to increase the sample size from this treatment for the welfare indicators that required sampling of individuals: the novel tank diving test, breeding trials, and neurotransmitter assay. The tanks were placed on three shelves in varying order of holding density (Figure 1). Each shelf held at least one tank per density treatment and all tanks had at least 25% individuals of either sex. The tanks were visually isolated from each other using a green-brown enrichment background depicting plants, and held one air stone and one floating green plastic plant each, in accordance with Stevens and colleagues (2021) [36].

The fish were fed 0.7% of their body mass (mean fish weight and SEM was 0.64 g +/− 0.016) of GEMMA Micro 300 pellets from Skretting (Stavanger, Norway) at 9 a.m. every morning according to the manufacturer’s recommendations. The feed was released at the water surface and spread across the entire tank, thus reducing the variability of food access for individual fish. The exposure lasted for nine weeks, between 24 November 2020, and 26 January 2021.

Our tanks were not connected to a sump system nor contained a bottom substrate, therefore we performed daily water changes (between 09:30 a.m. and 10:00 a.m.) in order to ensure no water interchange between the tanks. During the water changes, 1.8 L of water (corresponding to 60% of total water volume) was pumped out of the tank using an aquarium siphon, after which, the same volume of new water was added to the tanks. We prepared water for the daily water change three days in advance, allowing water to age properly with EasyStart bacteria culture (Easy-Life, Duiven, The Netherlands). This was done to ensure that the tank water contained a bacteria culture that could perform nitrification and ammonium removal despite the lack of surface area (e.g., bottom substrates or sump) for bacterial growth inside the tanks. Water samples from two randomly selected tanks were analysed twice each week for nitrates (NO_3_^−^-N), nitrites (NO_2_^−^-N), and ammonium (NH_4_^+^-N) concentrations, using LCK 339, 341, 304 test kits, respectively (Hach Lange, Dusseldorf, Germany). Throughout the exposure period, the mean bi-weekly concentration was 4.46 mg/L of NO_3_^−^-N, 0.25 mg/L of NO_2_^−^-N, and 0.38 mg/L of NH_4_^+^-N. To our knowledge, there are no specific guidelines on appropriate concentrations of nitrates for housing zebrafish. Other studies have found that nitrite levels should be kept below 73 mg/L (22 mg/L NO_2_^−^-N) to avoid adverse effects on the growth of adult zebrafish [37] and nitrate levels below 200 mg/L NO_3_^−^-N during the larvae period to avoid reduced growth and morphological abnormalities [38]. Our water values were considerably lower than these and are thereby concluded as acceptable by the limited research available. Other water parameters were recorded daily prior to water change, and pH was maintained at 7.1–7.7, temperature at 22.6–24.7 °C, and dissolved oxygen at 80–95% saturation. The conductivity of the water for water change was 680 µS and the pH was adjusted to 7.8. The photoperiod was kept at 14L:10D. The light in the room was on between 7:45 a.m., with a gradual increase until 8:00 a.m. and 21:45 p.m., with a gradual decrease until 22:00 p.m. 

All experimental procedures and husbandry protocols followed the ARRIVE guidelines [39] and the regulations set by the Swedish National Board for Laboratory Animals and were approved by the animal ethics committee in Gothenburg (#5.8.18-13943/2020). All fish were naïve to the experiments. 

### 2.2. Novel Tank Diving Test

The novel tank diving test was performed over a two-day period between 9 a.m. and 5 p.m., on all 374 fish in the experiment, according to the methods described by Landin and colleagues [40]. We chose to perform the experiments over two days to minimise the variation in exposure time between tanks. Individual fish were carefully netted from their exposure tanks and immediately released into the test aquarium while filmed. The tests were conducted using Aquaneering tanks (San Diego, CA, USA) with dimensions of 26.5 × 7.5 × 15 cm (L × W × H) and test water with the same pH, conductivity, and temperature as the exposure tanks. The fish were visually isolated from each other during testing by white walls and the tanks were washed and filled with new water between each test [41]. The tanks were divided into three depth zones at 0–4 cm (lower zone), 4–8 cm (middle zone), and 8–12 cm (upper zone) above the bottom. Recordings were made using two Sony video cameras (Tokyo, Japan) for 12 min while the fish explored the tank undisturbed. Each recording was subsequently analysed by tracking in EthoVision version 14.0 (Noldus, Wageningen, The Netherlands) for velocity, total distance moved, latency to the first entry of the middle and upper zones, the number of entries to the middle and upper zones, and cumulative duration of time spent in the lower, middle, and upper zones. Video analysis started when the fish entered the tank and stopped after 600 s. To avoid fish spending time in densities other than their assigned exposure density, we tested all fish from a single tank before proceeding to the next. In total, 358 analyses were successfully made with the tracking programme, *n* (1 fish/L) = 15, *n* (4 fish/L) = 35, *n* (8 fish/L) = 65, *n* (12 fish/L) = 104, *n* (16 fish/L) = 139. The remaining videos (*n* = 16) had poor quality and the software could not detect the fish for analysis of swimming movement. 

### 2.3. Vertical Distribution

The fish were automatically recorded for 30 min using three Foscam FI9851P wireless IP cameras (Egnir Invest, Son, Norway) at 2:00 p.m. and 5:00 p.m. during each day of the nine weeks of exposure. The recordings were pre-programmed and no one was allowed in the room during the recording time. Video analyses were made from the recordings for each day of the nine weeks. Each tank had a small tape-marking at the middle depth, thus creating two zones: upper half and lower half. In the analysis, the fish in each tank were scored together for their vertical distribution during 30 s of each recording, to ensure that the distribution was representative of the footage. A zone was scored if more than 50% of the fish were present in it. If the fish were not present in a majority in either of the two zones, a third option representing both zones was scored. In total, between 122 and 126 recordings were used to analyse the distribution in each tank, making a total of 1970 video scores across all tanks. The remaining 121 video scores had too poor of quality to detect a sufficient number of fish for analysis of vertical distribution.

### 2.4. Behavioural Observations

During the last eight days of the exposure period, the fish were observed by two trained scientists on seven occasions at randomised times of the day between 8:00 a.m. and 5:00 p.m. for a period of 10 min. The observations were conducted from a sufficient distance and angle so the fish could not see them. They observed each tank for chasing behaviour, where one or more fish were chasing each other, and hiding behaviour, where one or more fish were hiding in the back of the tank, behind the air stone, or the floating plastic plant. These behaviours were selected since they correspond to aggressive interaction in zebrafish [24]. Each tank received a score of one if the behaviour was present and a zero if the behaviour was absent. The scores for tanks holding the same density were added together and a percentage was calculated for how frequently the behaviour was observed (number of observations of behaviour/total number of observations). 

### 2.5. Breeding

After the novel tank diving test, the fish rested for 48 h in their exposure tanks before the breeding experiment. Zebrafish usually become sexually mature at the age of three months [42], so we expected all the six-month-old fish to be able to reproduce at this time. Breeding tanks were set up between 3:00 p.m. and 5:00 p.m., with one male and two females per tank, and left undisturbed to spawn until 12:00 p.m. the next day. Thereafter, the eggs were filtered and cleaned using clean tank water and transferred into graded 2 mL Eppendorf tubes from which the egg volume was measured. The eggs were then placed in Petri dishes where an experienced zebrafish scientist blindly determined the percentage of unfertilised versus fertilised eggs. The breeding trials were performed over a period of two days, with half of the exposure tanks being tested per day. A total of 48 breeding tanks were set up for all exposure tanks (Supplemental Table S1).

### 2.6. Cortisol Analysis

During the nine weeks, water was collected for cortisol analysis from each tank every Tuesday, beginning one week after the start of exposure. Water was always sampled between 2:45 p.m. and 3:15 p.m. to avoid variations caused by the circadian oscillation of cortisol [43]. Tank water was collected and stored in plastic jars made from polypropylene, a material to which cortisol does not bind. Between 3 and 10 dL of tank water was collected, poured into the jars, and immediately frozen at −20 °C until further analysis. Cortisol was extracted from the tank water according to Ellis and colleagues [44]. The water was peristaltically pumped using a Masterflex console drive (Cole-Parmer, Vernon Hills, Lake County, IL, USA) at 10 mL/min through a pre-filter (0.45 µm pore-size; Pall Corporation, Portsmouth, UK) and an extraction cartridge (Sep-pak^®^ Plus Short C18, Waters Ltd., Wilmslow, UK, activated with 2 mL methanol and 2 mL distilled water), washed with 5 mL of deionised water and frozen at −20 °C until elution. Free corticosteroids were eluted from the cartridge using 4 mL ethyl acetate, evaporated at 45 °C under nitrogen gas, dissolved in 100 μL standard diluent (phosphate buffer, NaCl, NaN_3_) [45], and frozen at −20 °C.

The radioimmunoassay was adapted from Young [46] and modified by Sundh and colleagues [45]. We modified the methods further by using 20 µL (instead of 10 µL) of extract from our samples. Our water cortisol levels were low, and this adjustment ensured that the concentrations were above detection levels in the scintillation counter. Additionally, we skipped the glutamate step described in these methods since the cortisol, being free in the water, was not bound to transport protein as it is in blood samples. The method adjustments were successfully tested prior to the sample analysis for validation. 

In short, a working stock was mixed using 9.9 mL standard diluent and 100 μL concentrated cortisol of 5 μg/mL prepared from hydrocortisone (Sigma-Aldrich, St. Louis, MO, USA). Using a standard diluent and the working stock, 11 cortisol standards (0.5 to 256 ng/mL) were prepared through serial dilution. Duplicate glass tubes were prepared with 20 µL of extract from each of the samples followed by the addition of 100 μL of working stock and 250 μL of radioactive corticosteroid (tritiated hydrocortisone-[1, 2, 6, 7–3H (N)], NEN Life Sciences Products, Boston, MA, USA; 15,000–25,000 disintegration per minute (dpm)) and cortisol antibodies (sheep anti-cortisol from Guildhay Ltd., Guildford, UK (no longer in activity), dilution: 1:3000). After vortexing, samples were incubated at room temperature for 120 min then placed in an ice bath for 5 min to stop the reaction. To remove unbound steroids, 200 μL charcoal-dextran solution was added to each tube. The samples were then vortexed and placed in a refrigerator for cooling for 25 min prior to centrifugation (3000 rpm, 4 °C, 15 min). The supernatants were poured into scintillation tubes, 5 mL scintillation fluid was added (Ultima gold, PerkinElmer, Waltham, MA, USA), and samples were vigorously mixed through shaking and vortexing. Radioactivity levels in the samples were analysed using a Wallac 1409 liquid scintillation counter (LKB Instruments, Turku, Finland), and the cortisol concentrations were calculated. To standardise the data and enable comparison between density treatments, the cortisol concentration for each tank was divided by the number of fish in the tank. To investigate how the cortisol concentrations per fish differed between the start of the experiment and the end of the experiments, we pooled the data from weeks 1 and 2 and then from weeks 8 and 9, for all tanks with a specific density and analysed for variations. 

### 2.7. Neurotransmitter Assays

After the breeding experiment, fish were returned to their exposure tanks and left to rest for 78 h. Thereafter, we conducted anaesthesia and euthanasia by rapid cooling (0–4 °C), decapitated the fish, and weighed all individuals. Heads and bodies were separated into different tubes, placed on dry ice, and stored at −80 °C. This was done within 20 s from anaesthesia to prevent the degradation of neurotransmitters. To quantify the brain content of neurotransmitters, we used high-performance liquid chromatography (HPLC). For the analysis, 60 heads (12 from each density) were randomly selected and thawed on ice and the whole brains were dissected on ice and weighed. Each brain tissue was homogenised in 100 µL of homogenisation solution made from 9.8 mL perchloric acid 0.1 M and 200 µL EDTA 10% using a Branson Ultrasonic Sonifier 250 (Fisher Scientific, Waltham, MA, USA). The samples were centrifuged at 12,000 rpm for 10 min at 4 °C and the supernatants were transferred to new tubes, followed by the addition of 10 µL of 10% trichloroacetic acid, vortexed, and centrifuged once more at 12,000 rpm for 10 min at 4 °C. Supernatants were transferred to a 1 mL syringe and filtered through a nylon syringe filter of 0.22 µm (Genetec, Montreal, QC, Canada) into new tubes. From here, samples were split to separately analyse monoamines and amino acids. For the monoamine transmitter analysis, samples were diluted at 1:2 with Milli-Q water. For the amino acid transmitter analysis, the samples were diluted at 1:100 with Milli-Q water. The monoamines were separated and detected using an HPLC with electrochemical detection (Dionex, Västra Frölunda, Sweden), as described by Clarke and colleagues [47]. To identify the monoamine peaks, external standards were used containing 3.25 fmol/μL for dopamine, 3.00 fmol/μL for serotonin, and 2.95 fmol/μL for norepinephrine. For the separation and detection of the amino acids, an HPLC with a fluorescence detector (Thermo Fisher Scientific, Gothenburg, Sweden) was used as previously described in Ulenius and colleagues [48]. Two external standards containing 0.5 µM and 1.0 µM of the amino acid arsenal were used for the identification of the amino acid peaks. 

### 2.8. Statistical Analysis

All statistical analyses were performed in SPSS (IMB SPSS Statistics for Windows, Version 24.0, IMB Corp, Armonk, NY, USA). Normal distribution was tested by considering histograms of the data sets and by the Shapiro-Wilk test. A *p*-value below 0.05 was considered statistically significant. 

The novel tank diving test parameters velocity, distance moved, and cumulative duration did not follow a normal distribution and were therefore analysed with non-parametric Kruskal–Wallis tests (KW). Latency of first entry to middle and upper zones was analysed using Kaplan Meier estimators (KM) with survival set as an event occurring before 600 s (total duration of the test) and log-rank tests. Frequencies of entries to each zone were analysed using negative binomial regression model (NBRM) analyses. 

The reproductive parameters egg volume and proportion of fertilised eggs did not follow a normal distribution and were analysed using KW tests. The whole-brain monoamine data followed a normal distribution and were analysed using one-way ANOVAs. For the cortisol analysis, the data met the assumption of normal distribution following a log transformation. The values from each week were therefore analysed for differences between treatments using one-way ANOVAs. Statistically significant one-way ANOVAs were followed by Tukey post hoc tests (alpha set to 0.05). To investigate the change in cortisol between the start and end of the exposure, a paired T-test was used (alpha set to 0.05). To assess the strength of association between holding densities and social behaviour, reproductive performance, and vertical distribution, respectively, we used odds ratios (OR) as a risk estimation with a 95% confidence interval (CI).

## 3. Results

### 3.1. Novel Tank Diving Test Showed That Anxiety Did Not Differ for Holding Densities between 1 and 16 Fish/L

**Figure 2:**
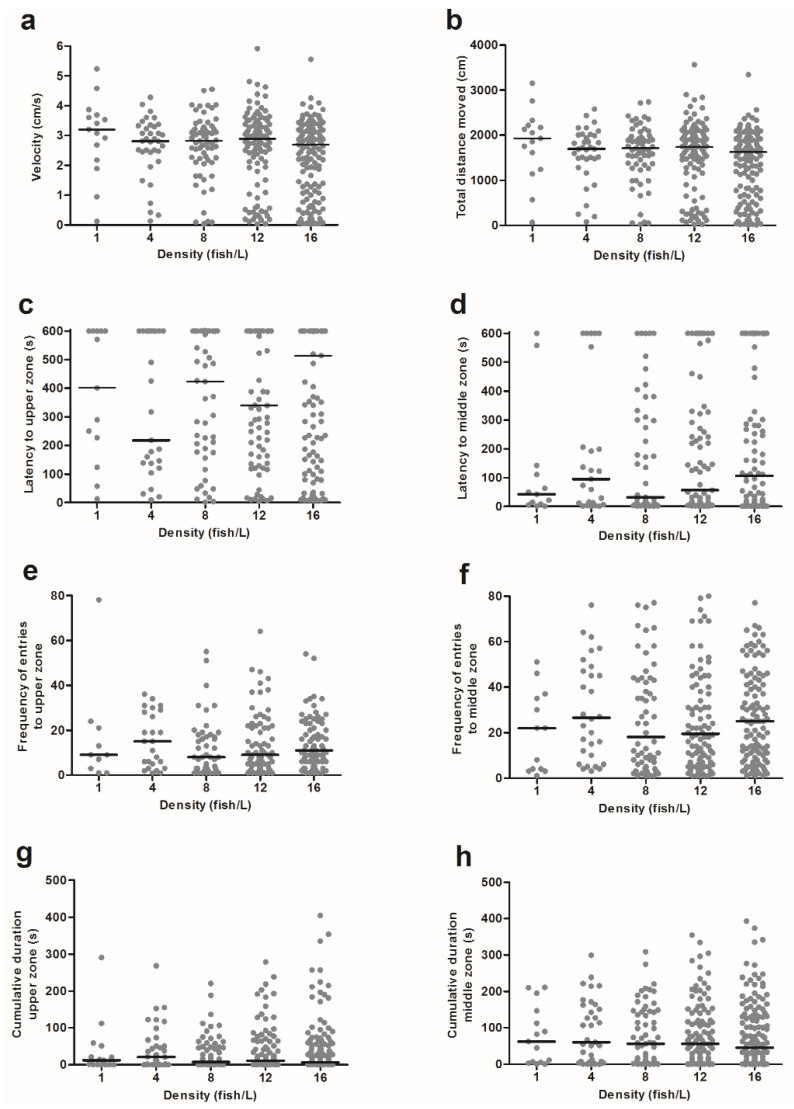
The behavioural responses in the novel tank diving test by zebrafish from holding densities of 1 (*n* = 15), 4 (*n* = 35), 8 (*n* = 65), 12 (*n* = 104), or 16 (*n* = 139) fish/L, respectively. Graphs show (**a**) velocity, (**b**) total distance moved, (**c**) latency to the upper zone, (**d**) latency to the middle zone, (**e**) frequency of entries to the upper zone, (**f**) frequency of entries to the middle zone, (**g**) cumulative duration in the upper zone, and (**h**) cumulative duration in the middle zone. Data points correspond to individual fish and medians are illustrated as lines. No statistically significant differences were found between the treatments. Each video analysis for the novel tank diving test lasted for 600 s.

The locomotion data from the novel tank diving test were analysed for total velocity, total distance moved, latency to the upper and middle zones, frequency of entries to the upper and middle zones, and cumulative duration in the upper and middle zones (Figure 2). All response variables of the different holding densities demonstrated similar medians and variances, thus indicating that there were no differences between the density treatments. 

There was no effect of the density on velocity (KW, H (4) = 6.544, *p* = 0.162), total distance moved (KW, H (4) = 6.953, *p* = 0.138), nor on latency to first entry to the upper zone (KM, χ^2^ (4) = 3.148, *p* = 0.533) or the middle zone (KM, χ^2^ (4) = 3.750, *p* = 0.441). Similarly, there was no effect on frequency of entries to the upper zone (NBRM, χ^2^ (4) = 3.532, *p* = 0.473) or the middle zone (NBRM, χ^2^ (4) = 2.016, *p* = 0.733), nor for cumulative duration in the upper zone (KW, H (4) = 1.361, *p* = 0.851), middle zone (KW, H (4) = 1.194, *p* = 0.879), or lower zone (KW, H (4) = 0.985, *p* = 0.912). Moreover, we stratified the data from the novel tank diving test for sex and reanalysed all variables. However, we did not find any significant differences in behavioural output between the sexes in this test (data not shown). Moreover, there was no effect of the time of the day when the novel tank diving tests were performed (data not shown). 

### 3.2. Fish Held at 1 Fish/L Were More Frequently Observed in the Upper Half of the Tank

**Figure 3:**
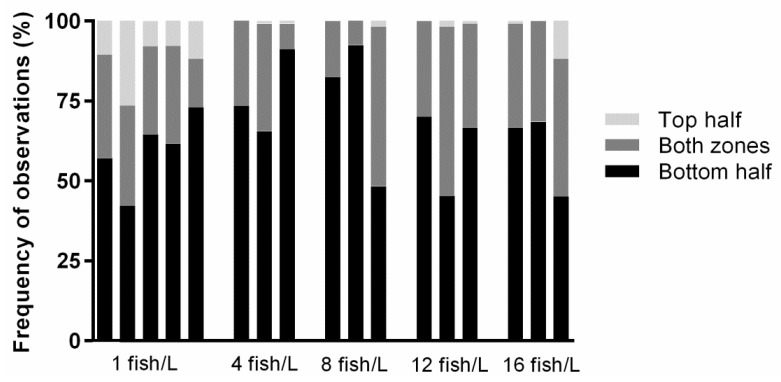
Vertical distribution for each tank holding densities of 1 (*n* = 5), 4 (*n* = 3), 8 (*n* = 3), 12 (*n* = 3), or 16 (*n* = 3) fish/L, respectively. There were three possible distribution zones: the bottom half of the tank, the top half of the tank, or both zones. The percentage frequency of observations was the number of observations during which 50% of the fish, or more, occupied one of these three zones. For each density treatment, there were three replicates and an additional two for the lowest density. Recorded videos of each tank were analysed twice a day for 30 s during the entire exposure period.

The overall most frequently observed vertical distribution was in the lower half of the tank, which was scored during at least 40% of the observations for all tanks (Figure 3). Fish were less often observed in the upper half of the tank, which was scored during less than 10% of the observations for most tanks (Figure 3). Holding density 1 fish/L was the only treatment where all tanks were observed to have fish occupying the upper half. Fish in the 1 fish/L treatment were more likely to be found in the upper zone compared to fish in the 4 fish/L treatment (OR = 0.059, 95% CI 0.019–0.190), 8 fish/L treatment (OR = 0.039, CI 0.009–0.159), 12 fish/L treatment (OR = 0.058, CI 0.018–0.187), and 16 fish/L treatment (OR = 0.306, CI 0.172–0.544). Otherwise, the fish in different tanks holding the same densities displayed large variations in how frequently they occupied the different zones. 

### 3.3. Aggressive Behaviours Occurred More Frequently at a Holding Density of 1 Fish/L

Chasing behaviour occurred most frequently (71% of the observations) in tanks holding 1 fish/L and decreased in frequency as density increased (Table 1). Similarly, hiding behaviour was most common in tanks holding 1 fish/L (63% of the observations). Fish in the 1 fish/L treatment were more likely to display chasing behaviour compared to fish in the 8 fish/L treatment (OR = 0.271, CI 0.087–0.839), 12 fish/L treatment (OR = 0.176, CI 0.054–0.574), and 16 fish/L treatment (OR = 0.104, CI 0.028–0.380) (Table 1). Fish in the 1 fish/L treatment were more likely to display hiding behaviour as opposed to fish in the 4 fish/L treatment (OR = 0.106, CI 0.026–0.427), 8 fish/L treatment (OR = 0.150, CI 0.042–0.538), 12 fish/L treatment (OR = 0.106, CI 0.026–0.427), and fish/L treatment (OR = 0.273, CI 0.085–0.877).

### 3.4. Reproductive Performance Was Similar at All the Tested Holding Densities

**Figure 4:**
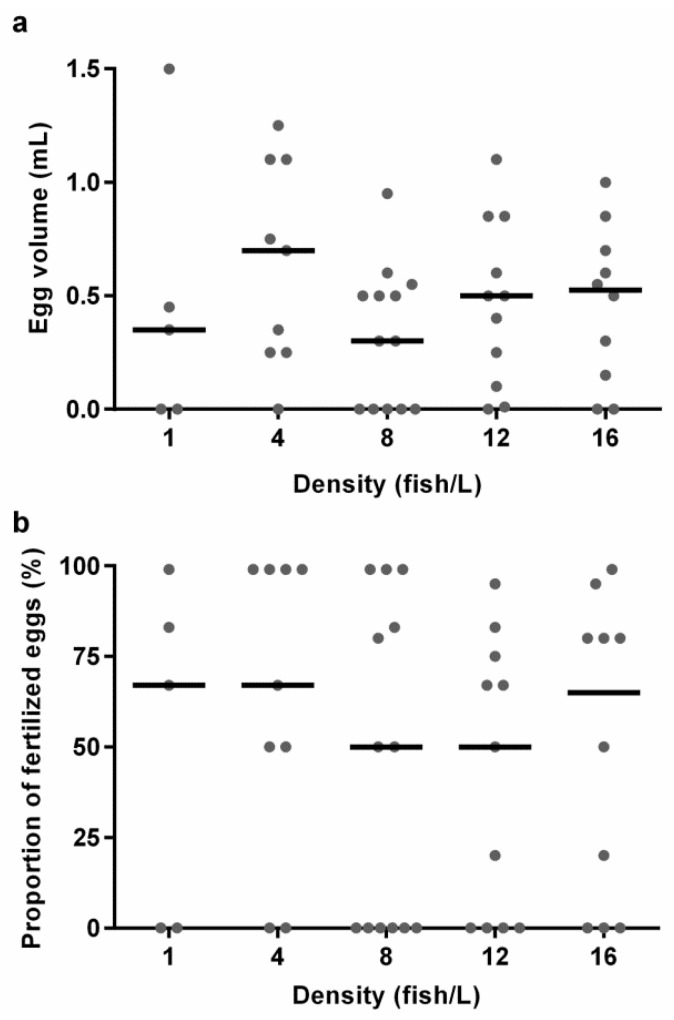
The results from the breeding experiments with zebrafish from holding densities of 1 (*n* = 5), 4 (*n* = 9), 8 (*n* = 13), 12 (*n* = 11), or 16 (*n* = 10) fish/L, respectively, from each breeding tank. Graphs show (**a**) egg volume and (**b**) proportion of fertilised eggs. Data points correspond to individual breeding tanks and medians are illustrated as lines. No statistically significant differences were found between the treatments.

There were large variations in the medians and variances for the different density treatments for both egg volume, the proportion of fertilised eggs, and fish spawning (Figure 4, Supplemental Table S1), thus indicating that holding density did not cause any detectable differences in reproductive output. There was no effect of the density treatment on egg volume (KW, H (4) = 3.406, *p* = 0.492), nor on proportion of fertilised eggs (KW, H (4) = 2.824, *p* = 0.588). The odds of fish spawning were the same in all densities (data not shown). 

### 3.5. Cortisol Secretion Was the Highest at a Holding Density of 1 Fish/L

**Figure 5:**
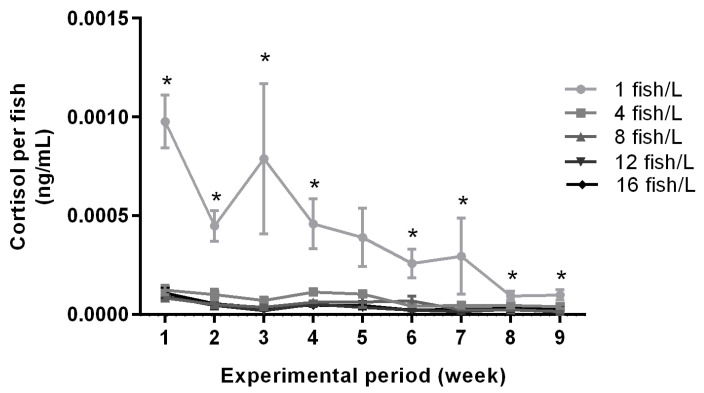
Water cortisol concentrations throughout the exposure period in tanks holding zebrafish at densities of 1 (*n* = 5), 4 (*n* = 3), 8 (*n* = 3), 12 (*n* = 3), or 16 (*n* = 3) fish/L, respectively. The graph shows cortisol levels per fish in each tank. Data are shown as mean ± standard error of the mean (SEM). Statistical significance between groups is presented as * *p* < 0.05.

The mean water cortisol concentrations per fish over time in the various holding densities are illustrated in Figure 5. There were significant effects of density treatment on cortisol concentrations for all nine exposure weeks (ANOVA, Supplemental Table S2). Tanks holding 1 fish/L had higher cortisol concentrations than all other treatments during exposure weeks 1, 2, 3, 4, and 6, higher than three other treatments for week 7, and higher than two other treatments for weeks 8 and 9 (Tukey tests, Supplemental Table S2). The differences in cortisol concentrations between treatments decreased over time (Figure 5). Cortisol values were significantly reduced from the first two weeks to the last two weeks in densities 1, 4, 8, 12 and 16 fish/L (*t*-test, *p* < 0.001, *p* = 0.001, *p* < 0.001, *p* = 0.003, *p* = 0.002, respectively). Moreover, there was no correlation between sex distribution in aquaria and cortisol secretion during the first exposure week (data not shown).

### 3.6. Whole-Brain Neurotransmitter Concentrations Did Not Differ between the Tested Holding Densities

**Figure 6:**
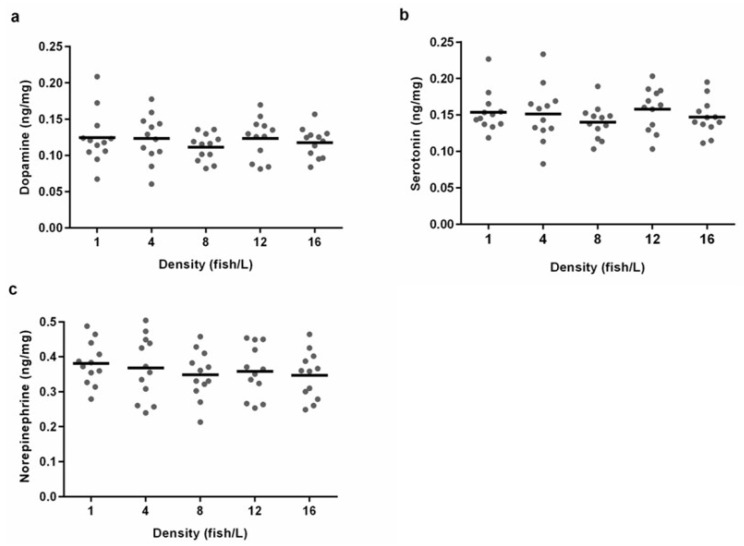
Whole-brain concentrations of monoamines for zebrafish from holding densities of 1 (*n* = 12), 4 (*n* = 12), 8 (*n* = 12), 12 (*n* = 12), or 16 (*n* = 12) fish/L, respectively. Graphs show individual concentrations of (**a**) dopamine, (**b**) serotonin and (**c**) norepinephrine. Means are illustrated as lines. No statistically significant differences were found between the treatments.

The whole-brain concentrations of monoamines dopamine, serotonin, and norepinephrine from fish in different holding densities had similar means and variances for zebrafish from all different density treatments (Figure 6). There was no effect of the density treatment on dopamine (ANOVA, F (4.55) = 0.471, *p* = 0.757), serotonin (ANOVA, F (4.55) = 0.644, *p* = 0.634), or norepinephrine (ANOVA, F (4.55) = 0.467, *p* = 0.759). We also performed an analysis of the whole-brain amino acids glutamate, serine, glycine, taurine, glutamine, and GABA, and found no statistically significant differences between the density treatments (Supplementary Figure S1).

## 4. Discussion

In short, our analysis of the majority of welfare indicators showed that the welfare was similar in all the tested holding densities. However, three welfare markers, i.e., cortisol release, vertical distribution, and aggression, singled out the lowest holding density of 1 fish/L from the other density treatments, suggesting increased stress and aggressive behaviour in this group. 

The present study, investigating the behavioural and physiological effects of different holding densities, has contributed to new findings regarding zebrafish welfare by using experimental methods that are novel to the zebrafish welfare field. Primarily, the experimental exposure period was long enough to allow us to study the animals’ welfare both during acute acclimatisation to the densities and chronic exposure to the treatment. In this way, we could follow behaviour and stress responses by continuous sampling throughout a nine-week exposure period. To the best of our knowledge, analyses of vertical distribution in the home tank, neither in response to holding density, nor over such a long period of time, have been performed previously. Secondly, using the novel tank diving test to assess the anxiousness of zebrafish in response to housing density has not previously been done [20]. Finally, our study analysed whole-brain neurotransmitters in relation to zebrafish holding densities, giving a deeper insight into the neurophysiological effects of zebrafish welfare and the neurobiology regulating anxiety behaviour. 

The novel tank diving test assesses the anxiousness of the fish and has been successfully used on zebrafish to assess both cognitive responses [27,49] and anxious behaviour in response to various drugs and stressors, such as alarm pheromones [50,51,52]. Our results from the novel tank diving test showed no detectable differences between the density treatments for any of the test parameters. This indicated that the anxiousness of the zebrafish was not affected by their holding density. 

Another important welfare indicator is reproductive success, especially considering that most laboratories, stock centres, and commercial dealers have continuous breeding programmes in place [53,54]. Since mating behaviour in zebrafish is a density-dependent process [55], the reproductive effects of various holding densities are important to evaluate. There were no detectable differences in total egg volume, nor the proportion of fertilised eggs, between the different holding densities. There were a number of breeding pairs from all treatments that did not lay any eggs (Supplemental Table S1). This was not surprising since all fish in the experiment were first-time layers and zebrafish usually require practice at reproducing in mating tanks before becoming successful breeders [56]. Previous studies evaluating the effects of zebrafish holding densities between 3 and 12 fish/L on various reproductive parameters found no differences between the treatments [57,58,59]. Rabbane and colleagues [59] tested holding densities between 5 and 45 fish/L and found that only densities above 25 fish/L had reduced reproductive output. Our results thereby agree with previous findings.

In our study, there were no detectable differences in whole-brain neurotransmitter concentrations, such as monoamines and amino acids, between the different density treatments. Previous research has found that chronic stress and social isolation can reduce brain concentrations of serotonin and dopamine in zebrafish [60,61,62]. The fact that the measured neurotransmitter levels were similar in all treatments suggested that the fish experienced the density treatments similarly.

In addition to monoamines, we measured free cortisol in the tank water. Cortisol is the primary stress hormone in zebrafish [52] and is known to increase in response to stressful situations, for example, acute crowding at densities of 40 fish/L [63,64]. Measuring water cortisol rather than whole-body cortisol has several advantages. Primarily, it is a non-invasive method that allows for multiple measurements from the same individuals in a tank, which can reduce the number of sacrificed laboratory animals. Secondly, research has shown that handling animals as short as 30 s prior to euthanasia can affect the whole-body cortisol response in the zebrafish [65], a problem that is avoided by measuring water cortisol. Finally, this method has been validated through positive correlations of whole-body cortisol and free water cortisol [66,67]. Therefore, we consider water cortisol to be an overall good alternative to whole-body cortisol measures, although one drawback of this method is that potential individual variation cannot be assessed and therefore might mask subtle differences between treatments.

Our results demonstrate that water cortisol concentrations per fish in tanks holding 1 fish/L had higher levels than all other treatments for the majority of analysed time points. These results signify that fish held at 1 fish/L have a higher stress level than fish in other holding densities, thus indicating reduced welfare at these low densities.

There are two previous studies on how holding density affects cortisol levels. Gronquist and Berges [68] found no differences in behavioural output and water cortisol for zebrafish over ten days when holding densities of 0.13 and 1.2 fish/L were compared, which were too low in relation to our tested densities for effective comparison. The second study by Pavlidis and colleagues [66] studied the effects of zebrafish holding densities of 4, 10, 20, or 40 fish/L, respectively, on trunk cortisol concentrations after fish were held at different densities for two hours. Similar to our results for the same density ranges, they did not find any differences in acute cortisol response. Overall, our cortisol results only single out the 1 fish/L density from others through elevated stress response to treatment. Egan and colleagues [52] found correlations between results in the novel tank diving test and whole-body cortisol measurements, where zebrafish displaying more anxious behaviour in the novel tank diving test had higher cortisol levels. Our study did not find this correlation, possibly due to Egan and colleagues [52] correlating individual behavioural and cortisol data, while our cortisol values were measured per tank. Moreover, at the time of anxiety testing, the differences in cortisol levels between 1 fish/L and the other densities were small (although significant) and this could also explain the lack of correlation with variables in the anxiety test.

It is very interesting that the results from the cortisol analysis single out the 1 fish/L treatment while the previously discussed welfare indicators did not. The cortisol levels in the tanks decreased with exposure time, and there are different possible reasons for this. Habituation is one, meaning that it takes time for zebrafish to habituate to a new social and physical environment. The 1 fish/L treatment would then serve as a more severe stressor to the fish compared to the other treatments as they displayed elevated cortisol levels for longer. Another possibility is that the long exposure period to a potentially stressful housing environment has created an allostatic load that caused a “wear and tear” on the hypothalamic-pituitary-interrenal (HPI) axis, which would then reduce the cortisol secretion over time [69]. A third possibility would be an increase in the metabolic clearance rate of cortisol in the circulatory system over time [69]. Although the hormone production may remain the same, this would result in less cortisol secreted from the gills of the fish to the surrounding water. However, considering that no other welfare indicator in this experiment showed stress for the 4–16 fish/L density treatments, habituation is the most likely explanation.

Water cortisol levels and vertical distribution were the non-invasive welfare indicators that were continuously measured during the exposure period. The remaining parameters were measured at the endpoint, so as not to interfere with the density treatments. It is possible that acute differences between treatments could have been found if tested earlier in the experiment, before the fish could habituate to their exposure density. In future research, it would be interesting to test the differences between acute and chronic treatments, either by increasing the length of the exposure period and continuing to evaluate the cortisol levels over time or by continuous sampling other welfare indicators, if possible, while considering their potential effects on the treatment. Nevertheless, our study contributes important data regarding how welfare can change over time, as there is a lack of previous studies that have focused on the long-term effects of holding densities as most have focused only on acute responses [20].

Vertical distribution was tracked for the entire exposure period as a new zebrafish welfare indicator to be evaluated. Previous studies have found that schools of other fish species alter their vertical distributions by moving closer to the bottom in response to stressors [21,22,23]. For zebrafish, previous research has found that vertical distribution can change in response to pain [70], during exposure to anxiogenic and anxiolytic drugs [71], and during exposure to alarm pheromones in certain populations [72]. To the best of our our knowledge, vertical distribution has not previously been studied in zebrafish as a welfare indicator, and we, therefore, wanted to explore this parameter.

Our results on vertical distribution displayed large variations between tanks holding the same, and different, densities for the 4 fish/L to 16 fish/L treatments. We found the most common distribution zone for all tanks to be the lower half, which agrees with observations of zebrafish during the control conditions investigated with this test (e.g., the study by Gerlai and colleagues [50]). In a study by Spence and colleagues [73], however, they observed captive zebrafish held along with other small fish species at a density of 0.08 fish/L in a large outdoor aquarium and found that zebrafish were equally distributed in all depth zones. Our closest tested density to theirs was 1 fish/L, which interestingly was the only treatment where all tanks had fish occupying the upper half, and thus had a vertical profile more similar to that reported in Spence and colleagues [73] compared to our other densities. Although their study set-up was not similar to ours, the data suggest that increased vertical distribution could be associated with low densities of 1 fish/L or below.

Since our results of vertical distribution singled out the 1 fish/L holding density, similarly to our results for water cortisol and social behaviour, we suggest that vertical distribution could be a useful non-invasive welfare indicator for zebrafish. We encourage further studies of zebrafish welfare to evaluate this non-invasive measurement.

Our results on vertical distribution could be a reflection of the social interactions observed during the last exposure week. Aggressive behaviour, indicated by chasing and hiding [24,25], occurred most frequently in the lowest density treatment and was reduced as the holding densities increased. Previous studies have found increased holding density to both elevate and reduce aggression in zebrafish [68,74], thus both supporting and contradicting our results. These studies have, however, only tested densities below 1.2 fish/L, which complicates comparisons. Nonetheless, the hierarchical behaviours observed in the 1 fish/L density treatment suggest that the dominant fish chased the other fish away from certain areas of the tank and forced them to hide behind the air stone or floating plastic plant, which were located in the bottom and top zones, respectively. The scores of vertical distribution, where fish held at 1 fish/L were more frequently observed in the upper half of the tank, could thereby be a reflection of the aggressive behaviour seen in that density treatment. 

Dahlbom and colleagues [75] studied pairs of zebrafish that expressed hierarchical structures. They found that subordinate fish were more stressed than control or dominant fish, indicated by elevated serotonin levels in the brain. While we did not find such an agreement between observed aggressive behaviour and the whole-brain concentrations of neurotransmitters, we did find an association between observed behaviour and higher cortisol concentrations in tanks with 1 fish/L. The elevated cortisol levels could therefore be explained by increased aggressive behaviour and social hierarchies. 

It might seem surprising that the welfare indicators of fish held at 16 fish/L were similar to those at lower densities. The welfare indicators collected after the treatment period of nine weeks in our study indicate that after a time of adjustment to the new holding conditions, the welfare is good at densities that could normally be considered “high”, according to previous density studies [20]. The reasons for zebrafish welfare being unaffected by these densities could be many, including the fact that the water quality was monitored and adjusted daily, and that the amount of feed was properly adjusted to the number of fish per tank. This is supported by a previous study on Atlantic salmon (*Salmo salar L.*) that found the cause of stress at elevated holding densities was more likely due to poor water quality (indirectly linked to high densities) than high densities, per se [16]. It is also possible that other welfare indicators, not evaluated in our study, would have identified differences between the treatments. Since the literature on the welfare effects of different zebrafish holding densities is sparse [20], future research should be conducted in similar experimental designs, but evaluating other welfare parameters. 

Moreover, one interesting explanation for the potentially low stress levels at higher densities could also be that since the fish tanks were not connected to each other via a common water flow, signalling molecules such as cortisol and other substances were not shared between individual tanks, which could otherwise have affected the stress levels of the fish. However, similar experiments have not yet been performed comparing isolated and recirculating systems to explore if recirculating signalling molecules could affect zebrafish welfare. This possibility would be of importance when designing filtering systems on current holding racks used by the zebrafish community.

Finally, the zebrafish is a social species, supported by density data from the wild [76], and social enrichment by peers could therefore be an important factor to ensure good welfare. Bearing in mind that the tanks used here (3 L) are the most commonly used tanks for zebrafish housing, this aspect should be taken into consideration as low holding densities would mean few fish per tank and thereby less social enrichment. 

## 5. Conclusions

In conclusion, our study shows that adult zebrafish can be housed at densities between 4 fish/L and 16 fish/L without causing adverse effects reflected by the welfare indicators measured, including anxiousness in the novel tank diving test and vertical distribution, reproductive success, whole-brain neurotransmitter concentrations, water cortisol concentrations, or social interactions through chasing and hiding behaviour. The results also suggest that laboratories should avoid housing zebrafish at the density of 1 fish/L since it caused increased aggressive behaviour, thus negatively affecting their welfare. Additionally, the elevated water cortisol levels indicate that fish in this treatment experienced more stress than others. The regulations and advice for zebrafish from organisations and governmental agencies, such as the Swedish Board of Agriculture, recommending keeping fish at low densities, such as 1 fish/L, should therefore be revised accordingly. According to our study, housing zebrafish at 4 fish/L to 16 fish/L is preferable since it does not compromise fish welfare. Future research is needed to determine the upper limit for holding densities. 

## Figures and Tables

**Table 1:** Behavioural observations from the last week of the exposure period. Each tank in the setup was given a score, once per observation, corresponding to one if the behaviour was displayed or zero if the behaviour was absent. The results presented here show the average percentage during which a behaviour was displayed for tanks holding the same density.

Behaviour (% of Observations)	1 Fish/L	4 Fish/L	8 Fish/L	12 Fish/L	16 Fish/L
Chasing	71	52	38	24	21
Hiding	63	14	24	14	36

## Data Availability

The data presented in this study are available on request from the corresponding author.

## Supplementary Materials

The following supporting information can be downloaded at: https://www.mdpi.com/article/10.3390/biology11050725/s1, Table S1: Results of the ANOVA and Tukey post hoc tests for cortisol levels per fish in each tank; Table S2: The proportion of successful breeding pairs in the reproductive trials for zebrafish from holding densities of 1, 4, 8, 12, or 16 fish/L; Figure S1: Whole-brain concentrations of amino acids for zebrafish from holding densities of 1, 4, 8, 12, or 16 fish/L.
