# Supplementary material for: Low Holding Densities Increase Stress Response and Aggression in Zebrafish
Source: Biology (Basel). 2022 May 9;11(5):725. doi: 10.3390/biology11050725 (PMC9139139; doi:10.3390/biology11050725)
Supplement: Supplementary file 1 [file biology-11-00725-s001.zip › biology-1713405-supplementary.pdf]

Low holding densities increase stress response and aggression in zebrafish

Marica Andersson, Jonathan A. C. Roques, Geoffrey Mukisa Aliti, Karin Ademar, Henrik Sundh, Kristina Sundell, Mia Ericson, and Petronella Kettunen

SUPPLEMENTAL INFORMATION

Supplemental Table S1. The proportion of successful breeding pairs in the reproductive trials for zebrafish from holding densities of 1, 4, 8, 12, or 16 fish/L

**Density (fish/L)**	**Number of breeding tanks**	**Proportion of successful breeding trials (%)**
1	5	60
4	9	89
8	13	62
12	11	82
16	10	80

Supplemental Table S2. Results of the ANOVA and Tukey post hoc tests for cortisol levels per fish in each tank (data presented in Figure 5).

**ANOVA**				
**Week**	**Degrees of freedom between groups**	**Degrees of freedom within groups**	**F-value**	**p-value**
**1**	4	12	45.403	< 0.001
**2**	4	12	16.506	< 0.001
**3**	4	12	19.153	< 0.001
**4**	4	12	19.642	< 0.001
**5**	4	12	3.658	0.036
**6**	4	12	13.718	< 0.001
**7**	4	12	6.068	0.007
**8**	4	12	4.747	0.016
**9**	4	12	6.426	0.005
**Tukey post hoc tests**				
**Week**	**p-value 1 fish/L vs. 4 fish/L**	**p-value 1 fish/L vs. 8 fish/L**	**p-value 1 fish/L vs. 12 fish/L**	**p-value 1 fish/L vs. 16 fish/L**
**1**	< 0.001	< 0.001	< 0.001	< 0.001
**2**	0.009	< 0.001	< 0.001	0.001
**3**	0.006	< 0.001	< 0.001	< 0.001
**4**	0.009	< 0.001	< 0.001	< 0.001
**5**	NS	NS	NS	NS
**6**	0.007	0.032	< 0.001	< 0.001
**7**	NS	0.035	0.007	0.038
**8**	NS	0.029	0.029	NS
**9**	NS	0.004	0.002	NS

NS: not significant

Supplemental Figure S1

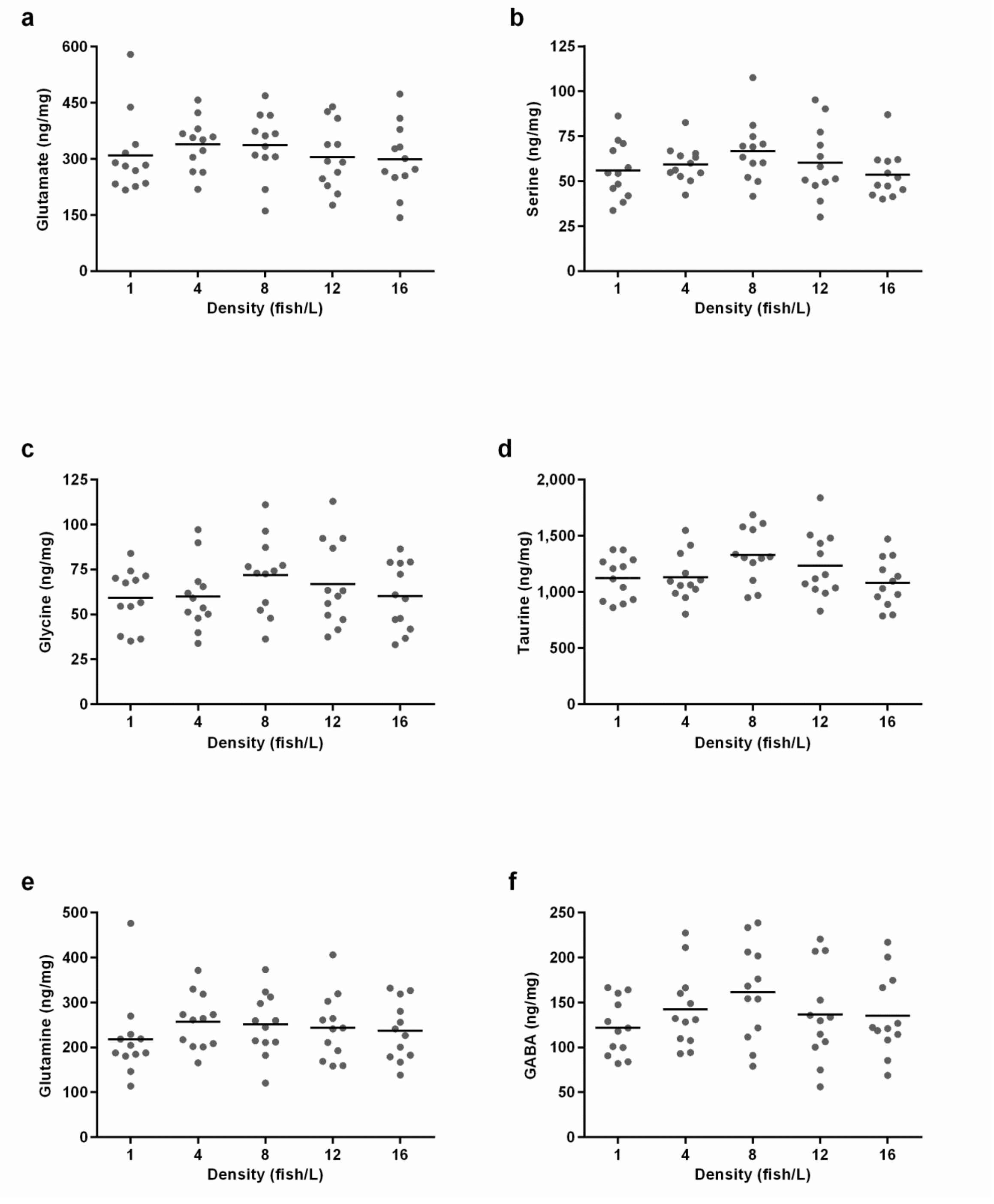

Supplemental Figure S1. Whole-brain concentrations of amino acids for zebrafish from holding densities of 1, 4, 8, 12, or 16 fish/L. Graphs show individual concentrations of (a) glutamate, (b) serine, (c) glycine, (d) taurine, (e) glutamine and (f) GABA. Means are illustrated as lines. One-way ANOVAs on the data demonstrated that there were no statistically significant differences between the density treatments for glutamate ($F_{(4,55)} = 0.538$, $p = 0.709$), serine ($F_{(4,55)} = 1.225$, $p = 0.311$), glycine ($F_{(4,55)} = 0.937$, $p = 0.450$), taurine ($F_{(4,55)} = 2.276$, $p = 0.073$), glutamine ($F_{(4,55)} = 0.508$, $p = 0.730$), or GABA ($F_{(4,55)} = 1.174$, $p = 0.333$).
